# P-644. Clinical and Sociodemographic Changes in Reported Pertussis Cases, Los Angeles County, 2014-2023

**DOI:** 10.1093/ofid/ofae631.841

**Published:** 2025-01-29

**Authors:** Chelsea Foo, Faith Washburn, Andrea Kim

**Affiliations:** Los Angeles County Department of Public Health, Los Angeles, California; Los Angeles County Department of Public Health, Los Angeles, California; Los Angeles County Department of Public Health, Los Angeles, California

## Abstract

**Background:**

In 2020, the pertussis national case definition was revised to classify any symptomatic PCR-positive case as confirmed, no longer requiring a cough illness of at least 14 days. The revision intended to improve case ascertainment, but the annual number of pertussis cases reported to Los Angeles County (LAC) Department of Public Health (DPH) has decreased since 2020 due to the pandemic. We sought to describe demographic and clinical differences among pertussis cases reported to LAC before 2020 versus 2020 onwards.

**Figure:**
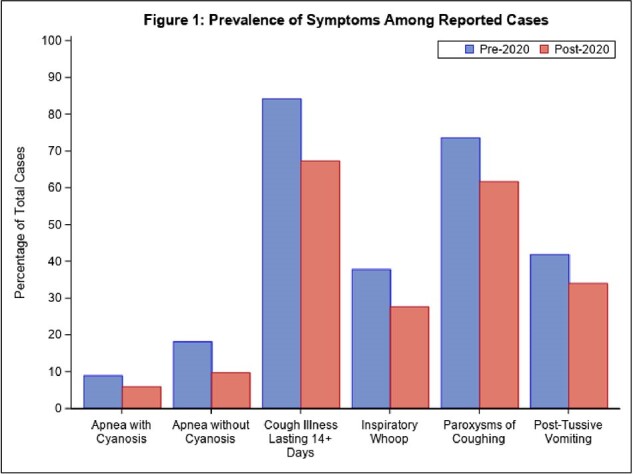

**Methods:**

All confirmed, probable, and suspect pertussis cases reported to LAC DPH from 2014 through 2023 were analyzed. Cases were categorized as pre-2020 (reported 1/1/2014 to 12/31/2019) or post-2020 (reported 1/1/2020 to 12/31/2023). Pearson’s chi-square test was used to assess bivariate associations between report period and case demographics and reported symptoms (paroxysms of coughing, inspiratory whoop, post-tussive vomiting, apnea with or without cyanosis, and cough illness lasting ≥14 days). A logistic regression model was built with report period as the outcome and symptoms and demographics as model covariates.

**Figure:**
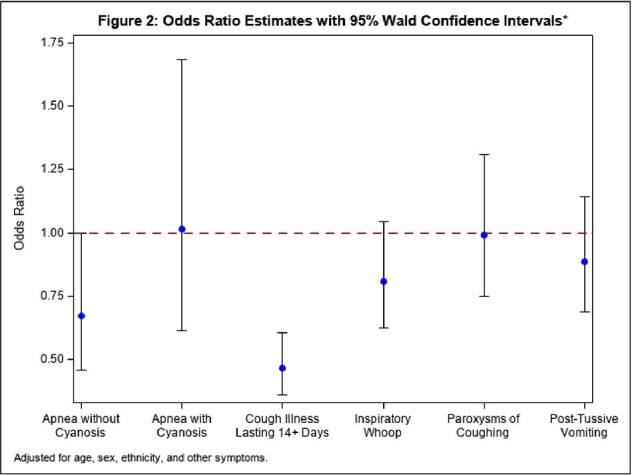

Event modeled is post-2020 with pre-2020 as reference group.

**Results:**

Of 6,594 cases reported, 391 (5.9%) cases were reported post-2020. Post-2020, the average annual case count decreased 91% from 1,034 to 98. Between time periods, case populations differed with respect to age and ethnicity (p< .001); sex remained similar (p=0.39). Post-2020, median case age decreased from 11 (interquartile range (IQR): 4-16) to 5 (IQR: 2-13). The prevalence of all symptoms decreased post-2020 (p< .001) (Figure 1). In the multivariable model, the association remained significant for cough illness lasting ≥14 days) (odds ratio (OR): 0.47 95% confidence interval (CI): 0.36-0.61) (Figure 2). This finding suggests that among those reporting cough illness lasting ≥14 days, the odds of diagnosis post-2020 versus pre-2020 were lower, after adjustment for age, sex, ethnicity, and other symptoms.

**Conclusion:**

The revised 2020 case definition may have increased case sensitivity in certain populations and among pertussis cases with less severe symptomology. Clinicians may consider PCR testing as soon as pertussis illness is suspected, which could enable sooner public health intervention and prevention of secondary transmission.

**Disclosures:**

**Chelsea Foo, MPH**, Assertio Holdings: Stocks/Bonds (Public Company)|Bionano Genomics: Stocks/Bonds (Public Company)|eHealth, Inc.: Stocks/Bonds (Public Company)|Johnson & Johnson: Stocks/Bonds (Public Company)

